# Supplementary material for: Protein Binder (ProBi) as a New Class of Structurally Robust Non-Antibody Protein Scaffold for Directed Evolution
Source: Viruses. 2021 Jan 27;13(2):190. doi: 10.3390/v13020190 (PMC7911045; doi:10.3390/v13020190)
Supplement: Supplementary file 1 [file viruses-13-00190-s001.pdf]

Protein binder (ProBi) as a new class of structurally robust non-antibody protein scaffolds for directed evolution

Phuong Ngoc Pham[#], Maroš Huličiak[#], Lada Biedermannová, Jiří Černý, Tatsiana Charnavets, Gustavo Fuertes, Štěpán Herynek, Lucie Kolářová, Petr Kolenko, Jiří Pavlíček, Jiří Zahradník, Pavel Mikulecky*, and Bohdan Schneider*

Institute of Biotechnology of the Czech Academy of Sciences, BIOCEV, CZ-25250, Vestec, Czech Republic

([#]) These authors contributed equally.

(*) Corresponding authors

Supporting Information

Table S1. List of primers.

Name	Sequence
ProBi-cloning-for	AAGTCCATGGCACAGGGACCCGGG
ProBi-cloning-rev	GTTCGGATCCGATGGAGCCCATGAATG
T7b	ATACGAAATTAATACGACTCACTATAGGGAGACCACAACGG
TolAk	CCGCACACCAGTAAGGTGTGCGGTTTCAGTTGCCGCTTTCTTTCT

Table S2. Data processing statistics and structure refinement parameters. Values in parentheses refer to the highest resolution shell.

PDB code	7AVC
Wavelength (Å)	0.91841
Space group	*H*32
Unit-cell parameters a, b, c (Å); α, β, γ (°)	72.7, 72.7, 192.8, 90.0, 90.0, 120.0
Resolution range (Å)	38.27 – 1.20 (1.22 – 1.20)
No. of observations	570,996 (14,200)
No. of unique reflections	59,704 (2,225)
Data completeness (%)	97.1 (74.4)
Average redundancy	9.6 (6.4)
Average $I/\sigma(I)$	5.4 (1.3)
R_{merge}	0.242 (1.106)
R_{pim}	0.117 (0.673)
$CC_{1/2}$	0.978 (0.498)
R_{work}	0.135
R_{free}	0.154
R_{all}	0.140
Average B-factor (Å^2)	17.0
RMSD bond lengths from ideal (Å)	0.012
RMSD bond angles from ideal (°)	1.638
Number of non-hydrogen atoms	1,403
Number of water molecules	203
Other molecules	2x GOL, 1x Na^+
Ramachandran statistics: residues in favored regions (%); number of outliers	100; 0

(A) pRDVsm casette

```
~TAATACGACTCACTATAGGGAGACCACAACGGTTTCCCAATAATTTTGTTTAACTTT
 T7 promoter           5′ stem-loop          RBS

AAGAAGGAGATATATCATATGGCATGGAGCCACCCGCAGTTCGAAAAGTCCATGGATA
               NdeI     Strep-tag II             NcoI

TGGAATTATTCGGATCCGAACAAAAGCTTATTTCTGAAGAGGACTTGGGATCTGGTGG
           BamHI c-Myc-tag

CCAGAAGCAA~
 TolA
```

(B) pETsm cassette

```
~TAATACGACTCACTATAGGGGAATTGTGAGCGGATAACAATTCCCCTCTAGAAATAA
 T7 promoter          lac operator

TTTTGTTTAACTTTAAGAAGGAGATATACATATGGCATGGAGCCACCCGCAGTTCGAA
 RBS                         NdeI     Strep-tag II

AAGTCCATGGATATGGAATTATTCGGATCCGAACAAAAGCTTATTTCTGAAGAGGACT
    NcoI                 BamHI c-Myc-tag

TGTAATAG~
  Stop codons
```

Figure S1. DNA sequences of internal arrangement within (A) the pRDVsm and (B) pETsm vectors. A modified version of pRDV or pET-26b vectors containing the N-terminal Strep-tag, short multi cloning site, and C-terminal c-Myc-tag with a stop codon.

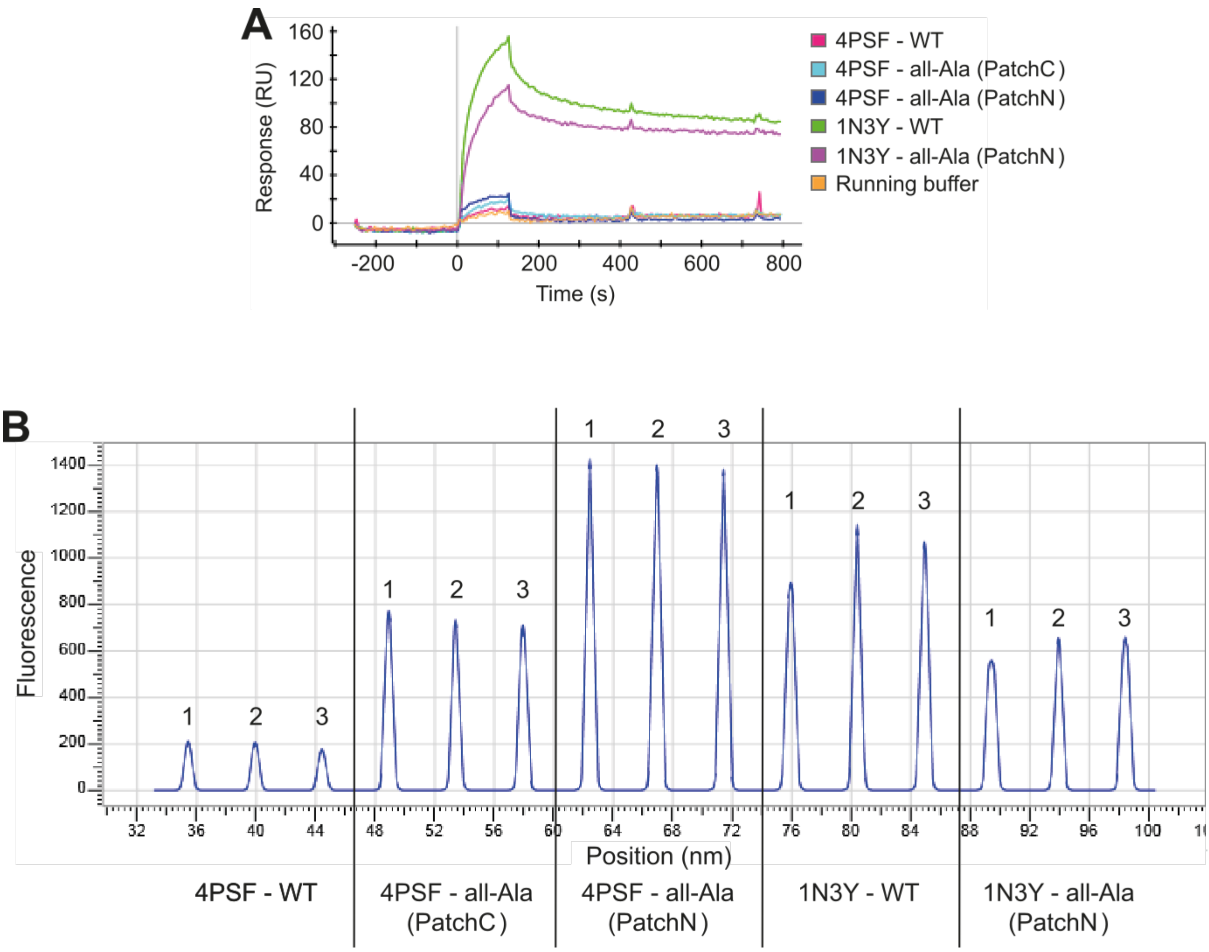

Figure S2. Testing of non-specific binding of scaffold candidates for (A) surface plasmon resonance (SPR) and (B) microscale thermophoresis (MST). Testing of SPR was performed on GLC chip. Testing of MST was done in three different types of capillaries – (1) Standard, (2) Hydrophilic, and (3) Hydrophobic.

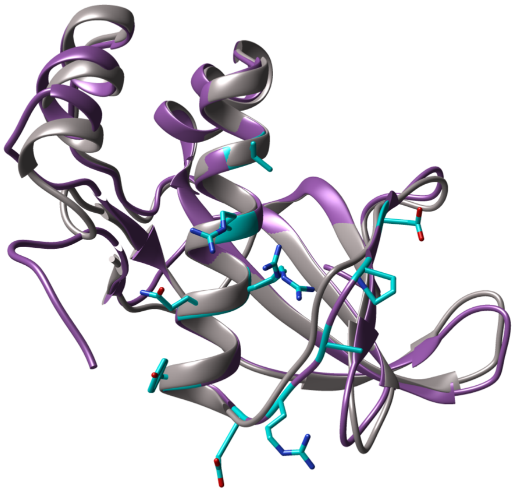

Figure S3. Structural alignment of the crystal structures of the J61 variant (violet ribbon) and the crystal structure of the WT (grey ribbon) of 4PSF. Mutated residues are highlighted in cyan and their side chains shown as sticks.

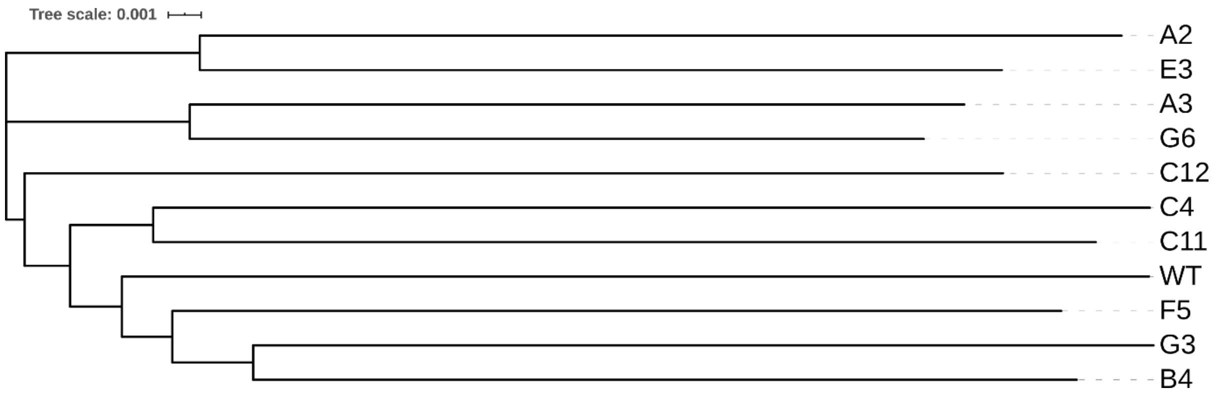

Figure S4. The phylogenetic tree of ten ProBi scaffold variants selected by ribosome display and used for more detailed biophysical characterization computed by multiple sequence alignment using EMBL-EBI Clustal Omega web service (www.ebi.ac.uk/Tools/msa/clustalo) with default parameters. Fasta-formatted sequences of the variants:

>WT
MAWSHPQFEKSMAQGPGQPGFCIKTNSSEGKVFINICHSPSIPPPADVTEEELLQMLEEDQAGFRIPMSLGEPHAELD
AKGQGCTAYDVAVNSDFYRRMQNSDFLRELVITIAREGLEDKYNLQLNPEWRMMKNRPFMGSIGSEQKLISEEDL
>A3
MAWSHPQFEKSMAQGPGQPGFCIKTNSSEGKVFINICHSPSIPPPADVTEEELLQMLEEDQAGFRIPMSLGEPHAELD
AKGQGCTAYDVAVNSDFYRRMQNSDFLRPLVIRIAVGGLERKYGLSLPPLWRMMKNRPFMGSIGSEQKLISEEDL
>G6
MAWSHPQFEKSMAQGPGQPGFCIKTNSSEGKVFINICHSPSIPPPADVTEEELLQMLEEDQAGFRIPMSLGEPHAELD
AKGQGCTAYDVAVNSDFYRRMQNSDFLRGLVIFIAVGGLESKYLLDLEPLWHMMKNRPFMGSIGSEQKLISEEDL
>C11
MAWSHPQFEKSMAQGPGQPGFCIKTNSSEGKVFINICHSPSIPPPADVTEEELLQMLEEDQAGFRIPMSLGEPHAELD
AKGQGCTAYDVAVNSDFYRRMQNSDFLRLLVILIAIVGLEWKYPLPLVPLWEMMKNRPFMGSIGSEQKLISEEDL
>C12
MAWSHPQFEKSMAQGPGQPGFCIKTNSSEGKVFINICHSPSIPPPADVTEEELLQMLEEDQAGFRIPMSLGEPHAELD
AKGQGCTAYDVAVNSDFYRRMQNSDFLRGLVIEIAPTGLEWKYFLLLEPSWCMMKNRPFMGSIGSEQKLISEEDL
>C4
MAWSHPQFEKSMAQGPGQPGFCIKTNSSEGKVFINICHSPSIPPPADVTEEELLQMLEEDQAGFRIPMSLGEPHAELD
AKGQGCTAYDVAVNSDFYRRMQNSDFLRVLVILIALLGLEVKYRLALQPVWYMMKNRPFMGSIGSEQKLISEEDL
>A2
MAWSHPQFEKSMAQGPGQPGFCIKTNSSEGKVFINICHSPSIPPPADVTEEELLQMLEEDQAGFRIPMSLGEPHAELD
AKGQGCTAYDVAVNSDFYRRMQNSDFLRGLVIRIAQRGIEFKYLLALNPRWIMMKNRPFMGSIGSEQKLISEEDL
>F5
MAWSHPQFEKSMAQGPGQPGFCIKTNSSEGKVFINICHSPSIPPPADVTEEELLQMLEEDQAGFRIPMSLGEPHAELD
AKGQGCTAYDVAVNSDFYRRMQNSDFLRRLVITIALRGLELKYPLCLRPAWHMMKNRPFMGSIGSEQKLISEEDL
>G3
MAWSHPQFEKSMAQGPGQPGFCIKTNSSEGKVFINICHSPSIPPPADVTEEELLQMLEEDQAGFRIPMSLGEPHAELD
AKGQGCTAYDVAVNSDFYRRMQNSDFLRRLVIAIAPNGLERKYTLHLTPTWSMMKNRPFMGSIGSEQKLISEEDL
>E3
MAWSHPQFEKSMAQGPGQPGFCIKTNSSEGKVFINICHSPSIPPPADVTEEELLQMLEEDQAGFRIPMSLGEPHAELD
AKGQGCTAYDVAVNSDFYRRMQNSDFLRGLVIGIAHRGLESKYYLRLGPRWWMMKNRPFMGSIGSEQKLISEEDL
>B4
MAWSHPQFEKSMAQGPGQPGFCIKTNSSEGKVFINICHSPSIPPPADVTEEELLQMLEEDQAGFRIPMSLGEPHAELD
AKGQGCTAYDVAVNSDFYRRMQNSDFLRRLVIRIARTGLELKYSLNLWPPWSMMKNRPFMGSIGSEQKLISEEDL

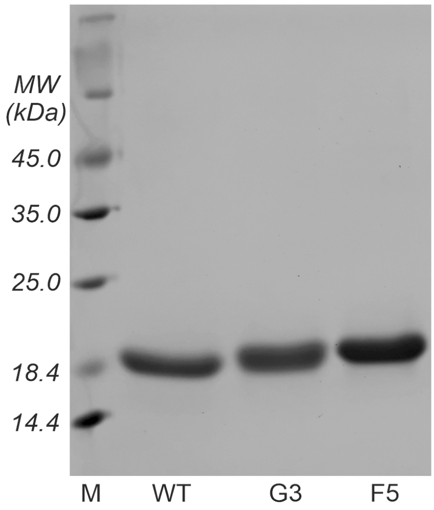

Figure S5. SDS-PAGE (15%) analysis of purified ProBi variants with the highest affinity measured by microscale thermophoresis (MST). We purified the proteins using the StrepTactinXT beads, followed by size exclusion chromatography.

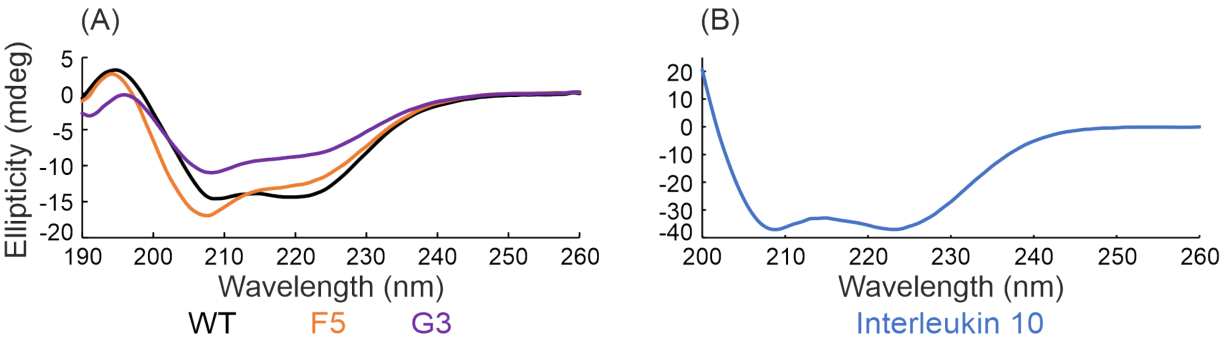

Figure S6. Circular dichroism spectra of (A) ProBi scaffold wild-type and two variants, and (B) interleukin-10. The CD spectra confirmed that all proteins were folded.

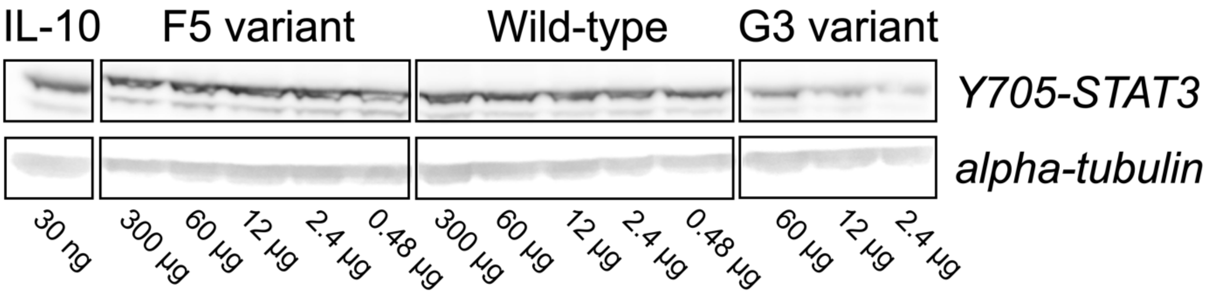

Figure S7. Inhibition of the IL-10 signaling pathway estimated by a competitive binding assay on the RAW264.7 cell line. The mixture contained IL-10 and a ProBi variant. The amount of IL-10 (30 ng) was the same in each well and the amount of ProBi proteins ranged from 300 µg to 0.48 µg. The ProBi variants with the highest affinity to IL-10, F5 and G3, showed no inhibition of the IL-10 signaling pathway on Western blot. We used the ProBi Wild-type protein as a negative control.
